# Signal Transducer and Activator of Transcription-3 Modulation of Cardiac Pathology in Chronic Chagasic Cardiomyopathy

**DOI:** 10.3389/fcimb.2021.708325

**Published:** 2021-08-24

**Authors:** Kristyn A. Hoffman, Maria Jose Villar, Cristina Poveda, Maria Elena Bottazzi, Peter J. Hotez, David J. Tweardy, Kathryn M. Jones

**Affiliations:** ^1^Department of Molecular Virology and Microbiology, Baylor College of Medicine, Houston, TX, United States; ^2^Department of Pediatrics, Section of Tropical Medicine, Baylor College of Medicine, Houston, TX, United States; ^3^Texas Children’s Hospital Center for Vaccine Development, Houston, TX, United States; ^4^Department of Biology, Baylor University, Waco, TX, United States; ^5^Department of Infectious Diseases, Infection Control & Employee Health, Division of Internal Medicine and Department of Molecular & Cellular Oncology, University of Texas MD Anderson Cancer Center, Houston, TX, United States

**Keywords:** STAT3, fibrosis, chronic Chagasic cardiomyopathy, inflammation, *Trypanosoma cruzi*

## Abstract

Chronic Chagasic cardiomyopathy (CCC) is a severe clinical manifestation that develops in 30%–40% of individuals chronically infected with the protozoal parasite *Trypanosoma cruzi* and is thus an important public health problem. Parasite persistence during chronic infection drives pathologic changes in the heart, including myocardial inflammation and progressive fibrosis, that contribute to clinical disease. Clinical manifestations of CCC span a range of symptoms, including cardiac arrhythmias, thromboembolic disease, dilated cardiomyopathy, and heart failure. This study aimed to investigate the role of signal transducer and activator of transcription-3 (STAT3) in cardiac pathology in a mouse model of CCC. STAT3 is a known cellular mediator of collagen deposition and fibrosis. Mice were infected with *T. cruzi* and then treated daily from 70 to 91 days post infection (DPI) with TTI-101, a small molecule inhibitor of STAT3; benznidazole; a combination of benznidazole and TTI-101; or vehicle alone. Cardiac function was evaluated at the beginning and end of treatment by echocardiography. By the end of treatment, STAT3 inhibition with TTI-101 eliminated cardiac fibrosis and fibrosis biomarkers but increased cardiac inflammation; serum levels of interleukin-6 (IL-6), and IFN−*γ*; cardiac gene expression of STAT1 and nuclear factor-κB (NF-κB); and upregulation of IL-6 and Type I and Type II IFN responses. Concurrently, decreased heart function was measured by echocardiography and myocardial strain. These results indicate that STAT3 plays a critical role in the cardiac inflammatory–fibrotic axis during CCC.

## Introduction

Chagas disease is caused by infection with the parasite *Trypanosoma cruzi* and currently affects an estimated 6–7 million people predominantly in Central and South America^1^. It is the leading cause of non-ischemic cardiomyopathy in Latin America (Bocchi, 2013). Acute infection is characterized as a non-specific febrile illness, with easily detectable parasitemia, that transitions to a clinically silent indeterminate phase with low to undetectable parasitemia (Dias et al., 1956). Overall, approximately 30% of infected individuals develop significant cardiac disease 10–15 years after infection, and approximately 2% of infected individuals develop chronic Chagasic cardiomyopathy (CCC) annually (Sabino et al., 2013). Mortality due to CCC is usually due to fatal arrhythmias, thromboembolism, or refractory heart failure (Rassi et al., 2010). Parasite-specific treatments, including benznidazole and nifurtimox, have been shown to reduce parasite burden but not to affect progression of cardiomyopathy (Morillo et al., 2015). The BENEFIT (Benznidazole Evaluation for Interrupting Trypanosomiasis) Study found that 17%–18% of patients with CCC progress to heart failure and death despite antiparasitic therapy (Marin-Neto et al., 2009; Morillo et al., 2015). Thus, treatment for patients with CCC also includes standard cardiac drugs to minimize arrhythmias and preserve heart function, including amiodarone, angiotensin-converting enzyme inhibitors, and aldosterone receptor agonists (Tanowitz et al., 2015; Nunes et al., 2018; Stein et al., 2018). However, improved understanding of the underlying mechanisms of disease progression in CCC will drive the development of more effective therapies.

The earliest cardiac histopathology findings in fatal cases of CCC were characterized by the persistence of parasites in the heart combined with diffuse fibrosis and inflammation in the myocardium (Dias et al., 1956). Further studies also identified autoimmunity, microvascular dysfunction, and dysautonomia as key factors in disease pathogenesis (Hyland et al., 2007; Bonney et al., 2019). In particular, the progressive cardiac fibrosis in CCC disrupts normal cardiac structural organization, resulting in worsening heart function, including arrhythmias and heart failure (Tassi et al., 2014; Tzizik and Borchardt, 2018). Transforming growth factor beta (TGF-β) is a master regulator of fibrosis and has been found to be elevated in CCC patients as well as preclinical models of CCC (Araujo-Jorge et al., 2002; Waghabi et al., 2002; Araujo-Jorge et al., 2008; Ferreira et al., 2016). Indeed, we recently demonstrated in our mouse model of CCC that TGF-β and downstream fibrosis mediators connective tissue growth factor (CTGF) and platelet-derived growth factor (PDGF) correlated with tissue fibrosis and worsening cardiac strain (Hoffman et al., 2019). Recently, inhibition of TGF-β was shown to decrease cardiac fibrosis and improve cardiac function in a mouse model of Chagas disease, indicating a key role of TGF-β in the mechanisms of fibrosis in CCC (Ferreira et al., 2019). However, additional investigation on the specific downstream targets of TGF-β will further define the key mechanisms of pathology in CCC and potentially identify new therapeutic targets.

Another classic lesion seen in CCC is chronic myocarditis that occurs as a result of the persistence of the parasite antigens in the heart. *T. cruzi* induces a pro-inflammatory response in which inflammatory cells infiltrate the myocardium, contributing to cardiac dysfunction (Andrade, 1999; Teixeira et al., 2002). Toll-like receptor signaling induces nuclear factor-κB (NF-κB)-mediated inflammatory signaling, leading to the release of pro-inflammatory cytokines (Andrade, 1999), e.g., interleukin-6 (IL-6) and interferon-γ (IFN-γ), as well as chemokines (Poveda et al., 2014). Transcriptome profile analysis of *T. cruzi*-infected cardiomyocytes revealed increased mRNA levels of cytokines and chemokines, including IL-6, a pleotropic cytokine that contributes to both inflammatory and fibrotic responses, depending on other disease factors (O’Reilly et al., 2014; Udoko et al., 2016; Kurdi et al., 2018). Importantly, IL-6 has been demonstrated to be responsible for macrophage polarization and subsequent myocardial damage in CCC (Sanmarco et al., 2017). Lymphocyte activation is amplified by IL-6 production induced by *T. cruzi* infection, leading to myocardial infiltration by T cells (Gao and Pereira, 2001). Importantly, IL-6 knockout (KO) mice infected with *T. cruzi* were shown to have reduced myocarditis compared to wild-type mice, confirming the role of IL-6 in *T. cruzi*-induced inflammation (Sanmarco et al., 2017). Furthermore, this suggests that IL-6 signaling may be a target for therapeutic intervention.

Signal transducer and activator of transcription-3 (STAT3) has been implicated as a key promoter of fibrosis development in animal models of fibrotic diseases (Ogata et al., 2006; Liu et al., 2013; O’Reilly et al., 2014; Pedroza et al., 2016; Su et al., 2017; Kurdi et al., 2018). STAT3 also has been found to be important in both IL-6 and TGF-β signaling and is directly activated by both IL-6 and TGF-β (Akira, 1996; Ogata et al., 2006; Fischer and Hilfiker-Kleiner, 2008; Hilfiker-Kleiner et al., 2010; Liu et al., 2013; O’Reilly et al., 2014; Tang et al., 2017). IL-6 binds its heterodimeric receptor consisting of IL-6Rα and IL-6Rβ (gp130), which induces the phosphorylation of the gp130-associated Janus kinase (JAK) and its subsequent phosphorylation of four tyrosine residues within gp130 (O’Reilly et al., 2013; O’Reilly et al., 2014). STAT3 binds to these phosphotyrosine (pY) peptide docking sites *via* its SRC homology 2 (SH2) domain resulting in its phosphorylation (O’Reilly et al., 2014; Johnson et al., 2018) at tyrosine 705 (pY-STAT3), which promotes its dimerization and accumulation within the nucleus, where pY-STAT3 homodimers drive pro-inflammatory and pro-fibrotic gene transcription (Johnson et al., 2018). STAT3 has been demonstrated to be activated by TGF-β through its heterodimerization with SMAD3 (Tang et al., 2017; Pedroza et al., 2018) resulting in upregulation of pro-fibrotic gene expression. Critical pro-fibrotic genes upregulated by activated STAT3 include Type I collagen, a key component of fibrosis (Papaioannou et al., 2018). Inhibition of STAT3 with TTI-101, a small molecule that targets the pY-peptide binding site within the SH2 domain of STAT3, has been found to ameliorate inflammation and fibrosis in the lung, skin, and liver (Xu et al., 2009; Pedroza et al., 2016; Pedroza et al., 2018). Importantly, cardiac-specific STAT3 signaling has been shown to play a critical role in inflammatory damage and age-related cardiac fibrosis (Jacoby et al., 2003). *T. cruzi* infection has been shown to increase STAT3 activation in the heart (Ponce et al., 2013). Together, these data suggest that STAT3 signaling plays a critical role in the pathogenesis of CCC.

Based on the findings summarized above, we hypothesize that STAT3 contributes to the chronic low-grade inflammation and progression of fibrosis seen in CCC and that use of TTI-101 may reduce cardiac fibrosis and/or inflammation in a mouse model of CCC. We have previously reported on our BALB/c mouse model of CCC that replicates the persistent inflammation and progressive fibrosis characteristic of human disease (Hoffman et al., 2019). The studies reported here were undertaken to investigate the consequence of STAT3 inhibition with TTI-101 on cardiac inflammation and fibrosis development in our animal model of CCC.

## Materials And Methods

### Parasites and Infections

*T. cruzi* H1 strain trypomastigotes, a DTU I strain originally isolated from a human patient with chronic cardiomyopathy in Mexico, were isolated from the blood of infected BALB/c mice [blood form trypomastigotes (bt)] at peak parasitemia [24–32 days post infection (DPI)] (Ruiz-Sanchez et al., 2005). Blood form trypomastigotes were washed once with sterile phosphate buffered saline (PBS), then resuspended in appropriate cell culture media (Complete Fibroblast Media). BALB/c mouse primary cardiac fibroblasts (Cell Biologics, IL, USA) were cultured in appropriate media and grown for 24 h prior to infection. Cell cultures were washed once with PBS, then infected with bt at a multiplicity of infection of 3 to achieve an infection efficiency of one parasite per cell. Cell culture medium was replaced every 24 h.

Wild-type BALB/c female mice (age 6–8 weeks), purchased from Taconic Biosciences, NY, USA, were injected intraperitoneally with 500 *T. cruzi* bt suspended in 0.1 ml sterile saline (n = 80) or sterile saline alone (n = 20) as previously described (Barry et al., 2016). Mice were evaluated daily for systemic signs of disease, including ruffled coat, lethargy, hunched posture, dyspnea, and visible weight loss. These signs were used as endpoint determinants, and mice that reached endpoint were humanely euthanized. All studies involving live animals were performed in strict compliance with Public Health Service Policy and The Guide for Care and Use of Laboratory Animals (Eighth Edition) and were approved by the Institutional Animal Care and Use Committee at Baylor College of Medicine under PHS assurance number D16-00475 (Council, 2011).

### Cardiac Parasite Burden and Parasitemia Quantification

Blood was collected from infected mice during the acute phase of disease, at 32 DPI, and hearts were collected at study endpoint (91 DPI). Total DNA was isolated from blood and heart tissue using a DNEasy 96 blood and tissue kit according to the manufacturer’s guidelines (Qiagen Sciences, MD, USA). Parasitemia and cardiac parasite burden were assessed by quantitative real-time PCR as previously described (Jones et al., 2018). Briefly, PCR was performed using 10 ng purified DNA, TaqMan Fast Advanced Master Mix (Life Technologies, CA, USA), and oligonucleotides specific for the satellite region of *T*. *cruzi* nuclear DNA (primers 5’ ASTCGGCTGATCGTTTTCGA 3’ and 5’ AATTCCTCCAAGCAGCGGATA 3’, probe 5’ 6-FAM CACACACTGGACACCAA MGB 3’; Life Technologies, CA, USA). Data were normalized to glyceraldehyde-3-phosphate dehydrogenase (GAPDH) (primers 5’ CAATGTGTCCGTCGTGGATCT 3’ and 5’ GTCCTCAGTGTAGCCCAAGATG 3’, probe 5’ 6-FAM CGTGCCGCCTGGAGAAACCTGCC MGB 3’; Life Technologies, CA, USA), and parasite equivalents were calculated from a standard curve of known parasite contents (Jones et al., 2018).

### TTI-101 Treatment

TTI-101, formerly known as C-188-9, a proven inhibitor of STAT3 phosphorylation (Xu et al., 2009), was acquired from Tvardi Therapeutics, Inc., TX, USA. For *in vitro* experiments, a stock of TTI-101 was prepared in dimethyl sulfoxide (DMSO), which was added to cell culture media to achieve a final concentration of 20 µM. For *in vivo* experiments, TTI-101 was dissolved in DMSO to achieve a concentration of 25 mg/ml and administered once daily to mice from 70 to 91 DPI by intraperitoneal injection of 0.05 ml for a dose of 1.25 mg/mouse or 62.5 mg/kg body weight.

### Benznidazole Treatment

Benznidazole (Laboratorio Elea, Argentina) was resuspended in 5% DMSO–95% HPMC (0.5% hydroxypropyl methylcellulose, 0.4% Tween 80, 0.5% benzyl alcohol in deionized water) to a final concentration of 10 mg/ml. Mice were treated with 100 mg/kg benznidazole by oral gavage once daily from 70 to 91 DPI. This treatment scheme was previously shown to reduce cardiac parasite burdens to nearly undetectable levels in BALB/c mice acutely infected with *T. cruzi* H1 (Cruz-Chan et al., 2021).

### STAT3 in Cardiac Cells Infected With *T. cruzi*

Cell cultures were treated with the compound after growth with and without *T. cruzi* infection. Cardiac fibroblast cells were grown for 24 h before infection to allow acclimation to culture conditions. All experimental groups were treated with a media change at this time, with the infected groups treated with three blood form *T. cruzi* Tc1 H1 trypomastigotes per cell, and the naive controls were treated with media alone. Cell lysate samples were collected at the following time points: 12, 24, 48, and 72 h. Protein in the lysate was quantified using a Pierce™ BCA Protein Assay Kit according to the manufacturer’s instructions (ThermoFisher, MA, USA), and all samples were diluted to 1 µg/µl. Protein isolated from lysate and cardiac tissue from mouse experiments (100 µg/well) was tested for concentrations of pY-STAT3 using ELISA (ThermoFisher, MA, USA). Samples were treated with RIPA Lysis and Extraction Buffer (ThermoFisher, MA, USA) for acquisition of lysate, according to manufacturer’s instructions. To test the effect of TTI-101 treatment on the levels of pY-STAT3 in cardiac fibroblasts, this experiment was repeated with TTI-101 treatment. Immediately after infection, the cell cultures were then treated with 50 µg/ml TTI-101, dissolved in DMSO, or DMSO alone. TTI-101 treatment was replenished at the same concentration with media changes every 24 h. Cell lysate samples were collected as previously, and lysates were tested for concentrations of pY-STAT3 using ELISA following the manufacturer’s instructions. Two technical and biological replicates were measured for each factor analyzed.

### STAT3 Inhibition With TTI-101 in a Mouse Model of Chronic Chagasic Cardiomyopathy

**Figure 1:**
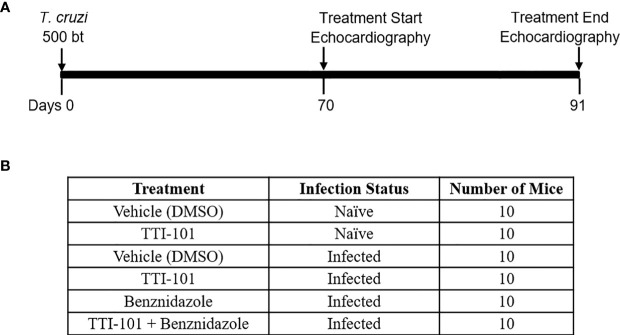
Experimental design. Shown is the timeline for experimental infection with *Trypanosoma cruzi*, TTI-101 and/or benznidazole treatment, and echocardiography imaging in a mouse model of chronic Chagasic cardiomyopathy **(A)**. Six treatment and control groups are listed, with untreated animals receiving dimethyl sulfoxide (DMSO) treatment vehicle alone **(B)**.

The infection and treatment timeline is illustrated in **Figure 1A**. At 70 DPI, mice were divided into six treatment groups, listed in **Figure 1B**. Prior to treatment start and end, at 70 and 91 DPI, all mice were anesthetized and imaged with echocardiography. Mice were immediately euthanized after the final echocardiography, and hearts and serum from the mice were collected for histologic analysis, gene expression, and ELISA analysis.

### Echocardiography

At 70 and 91 DPI, echocardiography was performed on anesthetized mice as described (Hoffman et al., 2019). Briefly, mice were anesthetized with inhaled isoflurane delivered by a precision vaporizer, and fur from the entire ventral thorax was removed with motor trimmers. Mice were positioned in dorsal recumbency on a temperature-regulated plate and monitored with rectal thermometer and Doppler ECG with four leads. The sternum was coated with a layer of ultrasound gel, and the left parasternal window was used to obtain both long and short axis echocardiogram images of the left ventricle in B-mode. Heart rates were maintained between 375 and 425 beats per minute during imaging acquisition. M-mode images were then obtained at the papillary level of the short-axis view in order to determine systolic and diastolic chamber dimensions.

The images were analyzed with Fujifilm Visualsonics VevoLab, Inc., Ontario, Canada echocardiogram analysis software. The left ventricle dimensions were obtained from the M-mode images to determine ejection fraction, stroke volume, and cardiac output. Each measurement represents the mean of 15 cardiac cycles of identical projection, transducer position, and angle of frozen images. Cardiac output values were normalized according to animal weight. The short-axis and long-axis left ventricle B-mode images were analyzed using the VevoStrain Fujifilm Visualsonics, Inc. Ontario, Canada software, with each measurement representing three cardiac cycles per position. The short-axis images were used to obtain circumferential strain. The long-axis images were used to obtain longitudinal strain.

### Histopathology Methods

At 91 DPI, all mice were anesthetized to a surgical plane of anesthesia with an intraperitoneal injection of ketamine (100 mg/kg) and xylazine (10 mg/kg), then euthanized *via* opening of the thoracic cavity. The heart was collected by excision and divided in half with sterile instruments. Then, one half was immediately fixed in 10% formalin solution and the other flash-frozen for later processing. The samples were dehydrated, embedded in paraffin, sectioned to 5 mm, fixed, and stained with hematoxylin–eosin (H&E) and Masson’s trichrome.

Imaging of the stained sections was performed on three representative images of the left ventricle in distinct regions that did not overlap. The imaging was performed with an Amscope United Scope LLC, CA, USA ME580 brightfield microscope equipped with LMPLAN40-065 ×40 objective using an 18-megapixel camera at fixed upper and lower light levels. ImageJ software was used to quantify the total area of the images that represented inflammatory infiltrate (H&E) or collagen (Masson’s trichrome). Two sequential sections of the left ventricle were imaged, with three representative images obtained from distinct non-overlapping portions of myocardium in each section, representing approximately 5% of the area per section. Briefly, for total fibrosis, numerical values of the total myocardium and the Masson’s trichrome-stained collagen area were obtained by adding the values of each area obtained with photography of ×40 microscopic fields in a section; the fraction of total area of collagen deposition per area of the entire section analyzed was calculated for each animal. Total heart area was quantified as the total area of the myocardium that appeared in each image, with white background subtracted from the total area. This same protocol was performed for quantification of area of inflammation using H&E staining.

### Immunohistochemistry

For immunohistochemistry (IHC) staining, mouse hearts were fixed in formalin, embedded in paraffin, and sectioned, as above. Heart sections were incubated with rabbit anti-CD4 monoclonal IgG (1:500; Cell Signaling Technology, Inc., MA, USA, 25229) or mouse anti-CD8 monoclonal IgG (1:50; Cell Signaling Technology, Inc., MA, USA, 98941) antibody followed by incubation with horseradish peroxidase (HRP)-conjugated secondary antibody. Brown color was carried out by incubation with the chromogen 3,3-diaminobenzidine (DAB), and nuclei were counterstained with hematoxylin.

### Cardiac Tissue Gene Expression Analysis

Frozen cardiac tissues were processed using Qiagen RNeasy kit to isolate and purify mRNA according to the manufacturer’s instructions. The mRNA was quantified and converted to cDNA using Qiagen RT2 First Strand kit. The cDNA was quantified with Nanodrop, and 20 μg was used in the Qiagen RT2 Profiler PCR Array Mouse Fibrosis kit with SYBR Green Rox Mastermix or NF-κB (Mm00476361_m1) and STAT1 (Mm00439518_m1) primers (ThermoFisher, MA, USA) with TaqMan™ Gene Expression Master Mix. Quantitative reverse transcriptase polymerase chain reaction (RT-qPCR) was carried out on an Applied Biosystems Viia 7 Real-Time PCR system. The relative quantitation of the genes was performed according to the ΔCt method using GAPDH as the internal control gene for individual primers and the housekeeping genes included in the mouse fibrosis array.

### Biomarker and Cytokine Targets and Analysis

To analyze the circulating levels of fibrosis biomarkers, inflammatory cytokines, and cardiac injury biomarkers, serum was collected from mice at 91 DPI. Blood was collected after transection of the descending aorta and caudal vena cava following euthanasia, and serum was collected from the blood. Concentrations of fibrosis biomarkers TGF-β and PDGF-D were measured using sandwich enzyme-linked immunosorbent assay (ELISA) kits according to the manufacturer’s instructions (Abclonal). Concentrations of inflammatory cytokines IL-6 and IFN-γ were measured using ELISA kits according to the manufacturer’s instructions (ThermoFisher, MA, USA). Protein extracted from cardiac tissue was analyzed for IL-6 with ELISA kit as well. Protein in the sample was quantified using a Pierce™ BCA Protein Assay Kit according to the manufacturer’s instructions (ThermoFisher, MA, USA), and all samples were diluted to 1 µg/µl for analysis of 100 µg/well. After completing kit instructions, all plates were analyzed with a Bioteck colorimetric plate reader for appropriate absorbance wavelength per assay instructions (450 nm). All absorbance values were normalized to blank wells and compared to standard curve for calculation of concentrations. Two technical replicates were measured for each factor analyzed.

### Statistical Analysis

All results are shown as mean ± standard deviation of two independent animal experiments, with two technical replicates. One representative experiment is expressed. Data were found to be normally distributed with the Shapiro–Wilk normality test. Two-way ANOVA and multiple-comparisons test were used when comparing multiple treatment groups to the naive control or untreated (infected) control. These tests were followed by Tukey’s honestly significant difference *post-hoc* test. When comparing two treatment groups to each other, a Student’s t-test was used. Analyses were performed using GraphPad Prism, Inc., CA, USA software, version 3.0. p ≤ 0.05 was considered statistically significant.

## Results

### TTI-101 Reduced pY-STAT3 in Infected Cardiac Fibroblasts

**Figure 2:**
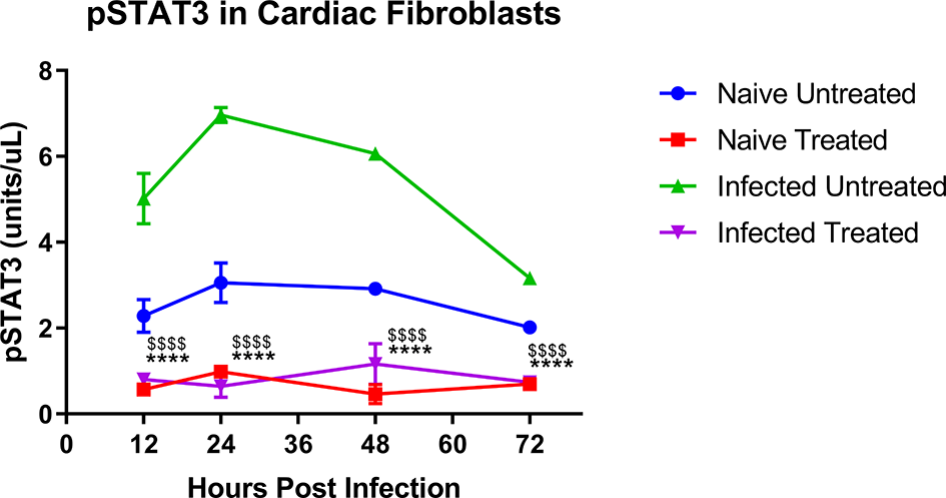
Active signal transducer and activator of transcription-3 (STAT3) concentration in cardiac fibroblast cell culture lysate. Shown are phosphorylated STAT3 (pY-STAT3) concentrations (units/µl, each µl contained 100 µg protein) in cardiac cell culture lysate. Separate cultures of cardiac fibroblasts were infected with one trypomastigote per cell of H1 TCI *Trypanosoma cruzi* strain or left naive. One each from the infected and naive groups was treated with TTI-101 (20 µM) or media alone. Cells were lysed at 12, 24, 48, and 72 h post infection. Cell lysates were analyzed with ELISA for pY-STAT3 concentration. Depicted are the summary data from two biological and two technical replicates. Error bars represent SD. Data were analyzed with two-way ANOVA; ****p < 0.0001 when comparing TTI-101-treated cells (purple symbols) to infected untreated cells (green symbols), ^$$$$^p < 0.0001 when comparing TTI-101-treated cells (purple symbols) to naive untreated cells (blue symbols).

The concentration of pY-STAT3 was significantly higher in untreated *T. cruzi-*infected cardiac (**Figure 2**, green symbols) fibroblasts than naive cells (**Figure 2**, blue symbols). TTI-101 treatment of infected cardiac fibroblasts (**Figure 2**, purple symbols) significantly reduced pY-STAT3 levels compared to infected untreated (**Figure 2**, green symbols) cardiac fibroblasts. Similarly, TTI-101 treatment of infected cardiac fibroblasts (**Figure 2**, purple symbols) significantly reduced pY-STAT3 levels compared to naive untreated cardiac fibroblasts (**Figure 2**, red symbols).

### Infection and Mortality

Treatment with TTI-101 was used to inhibit STAT3 activity, and treatment with benznidazole was used to clear the infection to evaluate the effect of STAT3 if the parasites were removed. Successful infection was measured by detecting *T. cruzi* parasites in the blood at 32 DPI. All animals in the infected groups were found to have positive parasitemia (**Supplemental Figure S1A**). Acute mortality was found to be 50% in the infected groups (**Supplemental Figure S1B**). The remaining mice that survived until 70 DPI were randomly assigned to groups of 10 mice each for treatment as described.

### Cardiac Parasite Burden and STAT3 Levels

**Figure 3:**
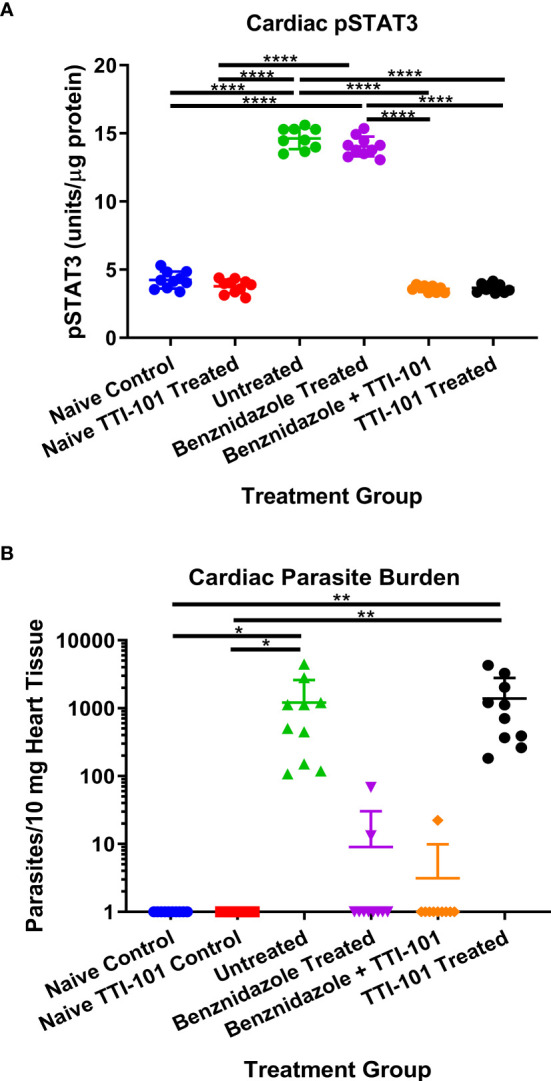
Cardiac parasite burden and signal transducer and activator of transcription-3 (STAT3) activity. Shown are the mean ± SD concentrations of pY-STAT3 measured in cardiac tissue **(A)** and the relative parasite burden per 10 mg cardiac tissue measured with qPCR at 91 days post infection **(B)**. Data were analyzed with Student’s t-test; *p < 0.05, **p < 0.01, ****p < 0.0001.

Cardiac levels of pY-STAT3 were quantified from all treatment groups to evaluate the effect of TTI-101 treatment on its target. The groups treated with TTI-101 alone and benznidazole with TTI-101 had significantly less pY-STAT3 in cardiac tissue than all other groups not treated with TTI-101, including the naive control group that received treatment vehicle alone (**Figure 3A**). The infected groups treated with drug vehicle or benznidazole alone had significantly higher levels of pY-STAT3 in cardiac tissue than all other groups.

Cardiac parasite burden was evaluated for the evaluation of infection status and effect of TTI-101 treatment on levels of parasites in the cardiac tissue. As expected, cardiac parasite burden was significantly reduced in both groups treated with benznidazole. Cardiac parasite burden in infected groups treated with TTI-101 alone or untreated was significantly greater than those in the naive controls and the groups treated with benznidazole with or without TTI-101 treatment (**Figure 3B**). Treatment with TTI-101 alone did not have any effect on cardiac parasite burden compared to vehicle treatment.

### Cardiac Fibrosis

**Figure 4:**
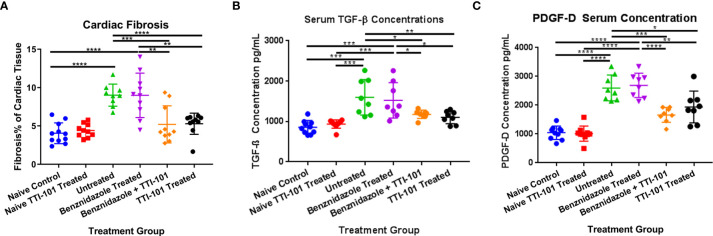
Cardiac fibrosis and fibrosis biomarkers in experimental animals. Relative fibrosis % of total cardiac tissue imaged is illustrated for 91 days post infection (DPI) at conclusion of treatment **(A)**. Terminal serum from all animals was collected at the conclusion of treatment at 91 DPI. Biomarkers of cardiac fibrosis were measured by ELISA. Serum concentrations of transforming growth factor beta (TGF-β) **(B)** and platelet-derived growth factor (PDGF)-D **(C)** at 91 DPI are shown. Representative images from one of two replicate experiments are shown. Error bars represent SD. Data were analyzed with Student’s t-test; *p < 0.05, **p < 0.01, ***p < 0.001, ****p < 0.0001.

To evaluate the effect of TTI-101 treatment on the development of cardiac fibrosis in chronically infected mice, cardiac fibrosis was quantified as the total percentage of Masson’s trichrome-stained collagen in the heart tissue of each animal in all groups. Infected groups treated with TTI-101 or benznidazole with TTI-101 had significantly lower levels of cardiac fibrosis than any other infected group (**Figure 4A**). Infected groups treated with treatment vehicle or benznidazole alone had significantly higher levels of cardiac fibrosis compared to all other groups. Cardiac fibrosis levels did not differ between the naive groups treated with treatment vehicle or TTI-101.

To evaluate the effect of TTI-101 on signaling involved in the development of cardiac fibrosis in CCC, serum concentrations of biomarkers of cardiac fibrosis, including TGF-β and PDGF-D, were measured. As expected, serum TGF-β was significantly increased in the serum of infected mice compared to naive controls, but TTI-101 treatment, with or without benznidazole, significantly reduced TGF-β levels (**Figure 4B**). Similarly, PDGF-D was significantly increased in the serum of infected mice compared to naive controls, but TTI-101 treatment, with or without benznidazole, significantly reduced PDGF-D levels (**Figure 4C**). Cardiac fibrosis biomarker concentrations were similar between the infected groups with TTI-101 or benznidazole with TTI-101 and both naive groups with treatment vehicle or TTI-101. However, pro-fibrotic biomarker levels in the infected groups treated with TTI-101 were significantly lower than both infected groups that were not treated with TTI-101.

### Cardiac Inflammation

**Figure 5:**
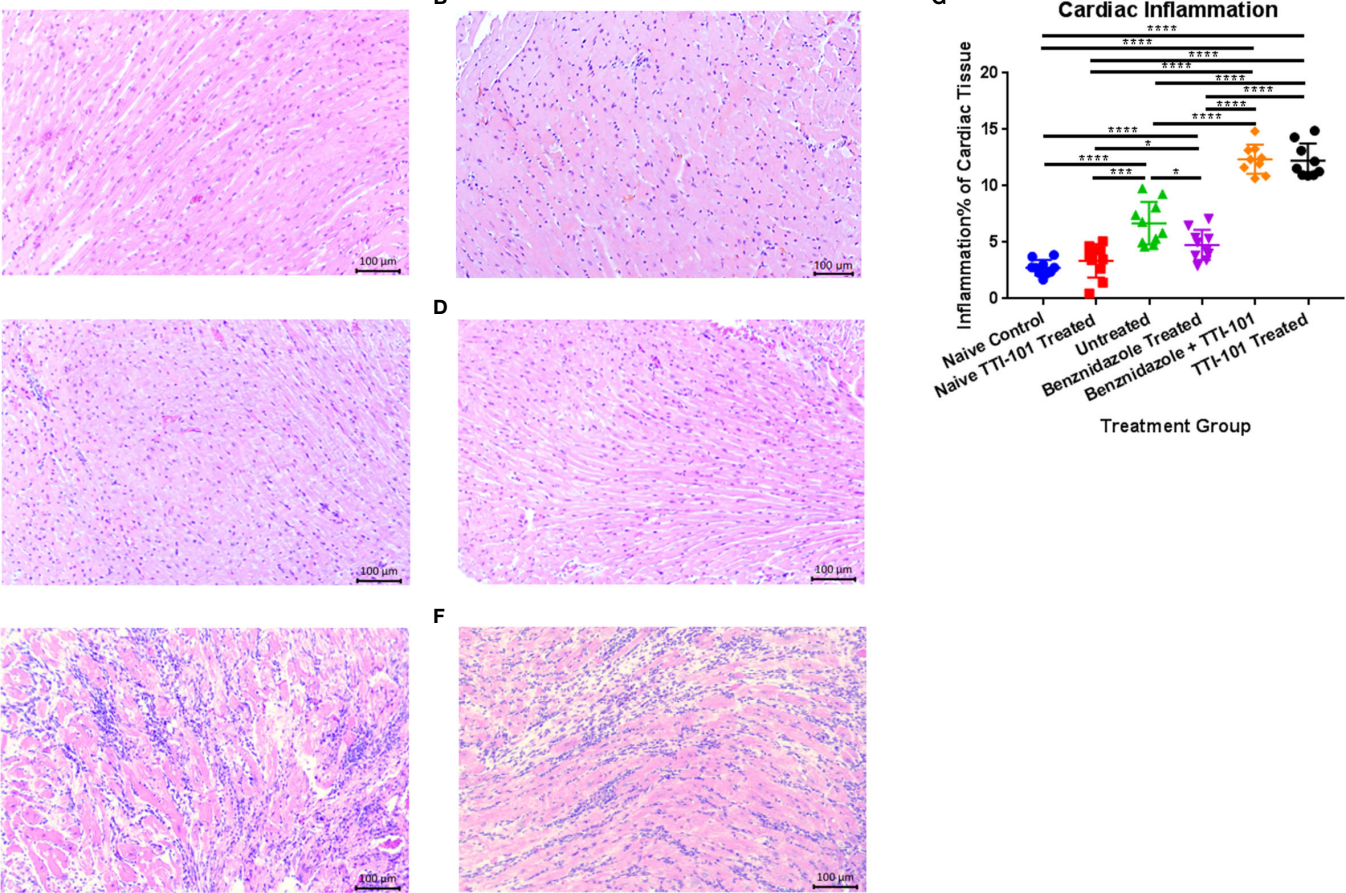
Cardiac inflammation quantified on histopathological analysis in experimental animals. Representative images of hematoxylin and eosin staining of infiltrating inflammatory cells in naive untreated **(A)**, naive TTI-101 treated **(B)**, infected untreated **(C)**, infected benznidazole treated **(D)**, infected TTI-101 treated **(E)**, and infected TTI-101 with benznidazole treated **(F)** are shown. Relative inflammation % of total cardiac tissue imaged is illustrated for 91 days post infection, at the conclusion of treatment **(G)**. Representative images from one of two replicate experiments are shown. Error bars represent SD. Data were analyzed with Student’s t-test; *p < 0.05, ***p < 0.001, **** p < 0.0001.

We have previously demonstrated that the *T. cruzi* H1 strain induces significant cellular infiltration into the heart in both acutely and chronically infected mice (Hoffman et al., 2019; Villanueva-Lizama et al., 2020; Cruz-Chan et al., 2021). Here, we used our established mouse model of CCC to evaluate the effect of TTI-101 treatment on cardiac pathology. Cardiac inflammation was quantified as a total percentage of the heart tissue on histopathology (Representative images, **Figures 5A–F**). Cardiac inflammation was significantly higher in groups treated with TTI-101 or benznidazole with TTI-101 than any of the other infected and naive groups and was reduced only in the infected group treated with benznidazole alone (**Figure 5G**). Cardiac inflammation was significantly higher in the infected groups receiving treatment vehicle or benznidazole treatment alone compared to both naive groups. Cardiac inflammation did not differ significantly between the naive groups.

**Figure 6:**
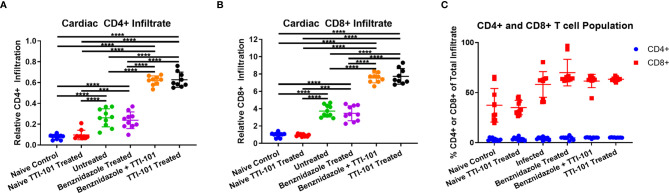
CD4 and CD8 staining of inflammatory infiltrate in cardiac tissue. Immunohistochemistry was performed on cardiac tissues from groups exhibiting myocarditis, and images were processed for the measurement of relative CD4+ and CD8+ populations. The relative CD4+ **(A)** and CD8+ **(B)** stained cells are depicted as a percent of total tissue, measured from heart samples from all treatment groups. Comparison of CD4+ and CD8+ percentages of total cellular infiltrate **(C)** is shown. Error bars represent SD. Data were analyzed with Student’s t-test; ***p < 0.001, ****p < 0.0001.

The inflammatory infiltrate was characterized with immunohistochemistry using antibodies for both CD4 and CD8. Staining for CD4 was detected in low levels in the inflammatory infiltrate of the hearts of any group with myocarditis (**Figure 6A**). However, inflammatory infiltrates stained positive for CD8 in all infected groups (**Figure 6B**). The groups treated with TTI-101 had greater levels of CD4+ and CD8+ infiltrate than all other groups (**Figures 6A–C**). However, the CD8+ proportion of inflammatory infiltrate was much greater than CD4+ for all infected groups (**Figure 6C**).

### STAT3 Inhibition Causes Increased Pro-Inflammatory Signaling Driven by IL-6 and IFN-γ

**Figure 7:**
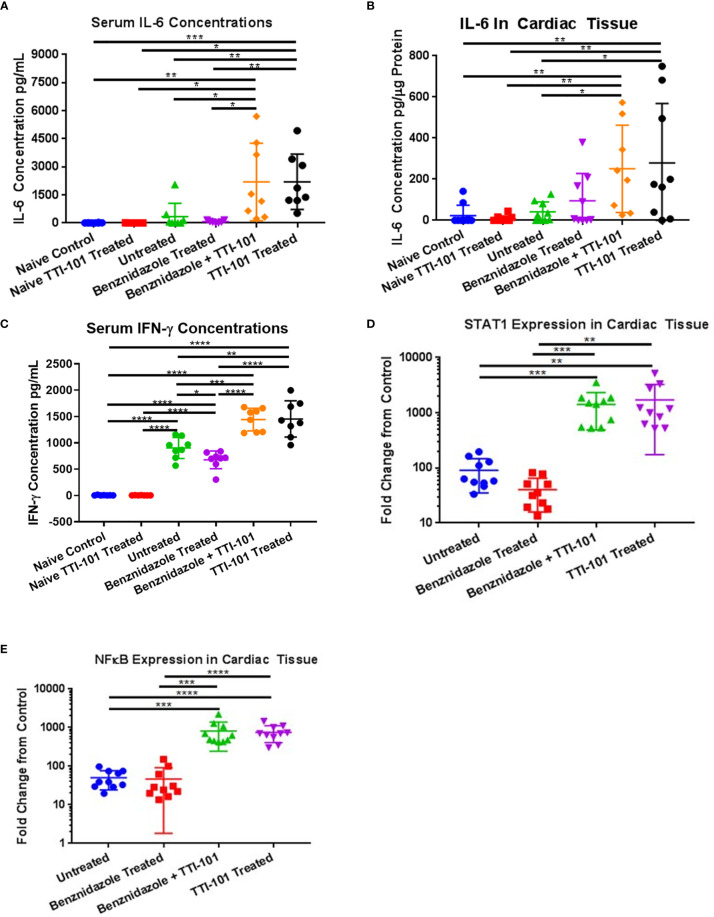
Inflammatory signaling induced by signal transducer and activator of transcription-3 (STAT3) inhibition. Pro-inflammatory signaling mechanisms influenced by STAT3 activity were measured in the serum and cardiac tissues of all animals after treatment with TTI-101. Shown are individual levels of serum interleukin-6 (IL-6) **(A)**, cardiac IL-6 **(B)**, and serum interferon-γ (IFN-γ) **(C)**. Downstream effectors of IL-6 and IFN-γ were measured in cardiac tissue. Cardiac gene expression levels of STAT1 **(D)** and NF-κB **(E)** are shown as fold increase above naive controls. Representative images from one of two replicate experiments are shown. Error bars represent SD. Data were analyzed with Student’s t-test; *p < 0.05, **p < 0.01, ***p < 0.001, ****p < 0.0001.

To characterize the signaling mechanism behind the increase in cardiac inflammation with STAT3 inhibition by TTI-101, serum and heart tissue were evaluated for evidence of pro-inflammatory signaling changes. To evaluate the effect of the inhibition of STAT3 activity on IL-6 pro-inflammatory pathway and IFN-γ pro-inflammatory pathway, concentrations of IL-6 were measured in serum and heart tissue, and serum IFN-γ levels were measured. Serum concentrations of IL-6 (**Figure 7A**) were significantly higher in groups treated with TTI-101 or benznidazole with TTI-101 than those in all other groups. Similar trends were seen in elevations of cardiac tissue IL-6 (**Figure 7B**), which, in combination with the serum data, suggest that local and systemic IL-6 responses are altered with TTI-101 treatment. Serum concentrations of IFN-γ (**Figure 7C**) were significantly higher in groups treated with TTI-101 or benznidazole with TTI-101 than those in all other groups. Additionally, serum levels of IFN-γ were significantly increased in the infected groups that were treated with treatment vehicle or benznidazole alone compared to the uninfected control groups.

To confirm that the downstream effectors in the IL-6 and IFN-γ pro-inflammatory signaling pathways were upregulated, gene expression levels of NF-κB and STAT1 were measured. All four infected groups had upregulated gene expression of STAT1 and NF-κB compared to uninfected controls (**Figures 7D, E** **)**. In groups treated with TTI-101 and benznidazole with TTI-101, gene expression levels of NF-κB and STAT1 were significantly higher than those in both the uninfected controls and the other infected groups.

### STAT3 Inhibition Reduces Cardiac Function Despite a Reduction in Cardiac Fibrosis

**Figure 8:**
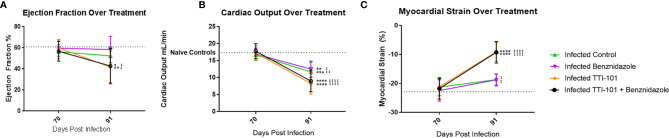
Cardiac function measured on echocardiography. Echocardiography was performed pre- and post-treatment to evaluate changes to cardiac function. All infected groups are compared to the naive untreated and naive TTI-101-treated groups (no significant differences were seen between the naive untreated and naive TTI-101-treated groups). Summary data measurements of ejection fraction **(A)** and cardiac output **(B)** measured by M mode echocardiography before and after treatment compared to naive control averages (dotted line). Summary data levels of myocardial strain of the left ventricle measured by B mode echocardiography before and after treatment compared to naive control average (dotted line; **(C**). Short-axis images were used to determine myocardial circumferential strain **(C)**. Representative images from one of two replicate experiments are shown. Error bars represent SD. Data were analyzed with Student’s t-test. When comparing groups to naive control, *p < 0.05, **p < 0.01, ***p < 0.001, ****p < 0.0001. For each group when comparing pre-treatment to post-treatment values ^±^p < 0.05, ^±±^p < 0.01, ^±±±±^p < 0.0001.

To evaluate whether heart function improved or declined in response to STAT3 inhibition during CCC, echocardiography was performed before and after treatment with TTI-101. There were no significant differences between ejection fraction levels among treatment groups before treatment (**Figure 8A**). Groups treated with TTI-101 and benznidazole with TTI-101 had a significant decrease in ejection fraction at the end of treatment compared to that before treatment. Additionally, the groups treated with TTI-101 and benznidazole with TTI-101 had a significantly lower ejection fraction than that in all other groups.

Before treatment, at 70 DPI, there were no statistically significant differences in cardiac output among the groups, including between the infected and naive groups (**Figure 8B**). At treatment completion, cardiac output was significantly reduced in all of the infected groups compared to baseline pretreatment measurements (**Figure 8B**). However, the TTI-101 treatment groups exhibited the greatest reduction in cardiac output. Moreover, those groups treated with TTI-101 exhibited the greatest myocardial strain alterations relative to the other groups, regardless of whether they were treated with benznidazole (**Figure 8C**).

We have previously shown that myocardial strain correlates with cardiac fibrosis in our mouse model of CCC (Hoffman et al., 2019). To evaluate the effect of STAT3 inhibition on myocardial strain, both longitudinal and circumferential myocardial strains [longitudinal and circumferential shortening expressed as negative % strain (Dandel et al., 2009)] were measured from echocardiography images. Before treatment, there was no significant difference in circumferential strain between any of the groups (**Figure 8C**). In groups treated with TTI-101 and benznidazole with TTI-101, myocardial strain significantly increased after treatment compared to that before treatment and was also significantly higher than that in all other groups after treatment. After treatment, the myocardial strain in the infected groups treated with treatment vehicle and benznidazole was significantly higher than that in the uninfected control groups.

## Discussion

Given previous evidence that signaling pathways involving STAT3 are altered in chronic fibrotic diseases and evidence showing altered STAT3 signaling in *T. cruzi* infected cells *in vitro*, we conducted the studies described here to determine the *in vivo* effect of inhibiting STAT3 on cardiac fibrosis development in CCC (Ponce et al., 2013; Kasembeli et al., 2018). To our knowledge, this is the first report to define the role of STAT3 in modulating the cardiac inflammatory–fibrotic axis in a mouse model of CCC. We confirmed that *T. cruzi* H1 infection significantly increased levels of pSTAT3 in cardiac fibroblasts *in vitro* and in heart tissue of chronically infected mice. We showed that similar to mouse models of skin, lung, and liver fibrosis (Pedroza et al., 2016; Kasembeli et al., 2018; Pedroza et al., 2018), inhibition of STAT3 signaling with the small molecule TTI-101 significantly reduced *T. cruzi*-induced pSTAT3 levels and tissue fibrosis. The reduced fibrosis was accompanied by significantly reduced serum levels of TGF-β and PDGF-D. Inhibition of STAT3 signaling also resulted in a significant increase in cardiac inflammation, with infiltration of both CD4+ and CD8+ cells into the heart, accompanied by increased expression of the pro-inflammatory transcription factors NF-κB and STAT1 and IL-6 protein levels. Concurrently, levels of IL-6 and IFN-γ were elevated in the serum. Inhibition of STAT3 signaling also resulted in decreased cardiac function, as evidenced by significantly reduced ejection fraction and cardiac output and worsening cardiac strain in mice treated with TTI-101. The effects of TTI-101 treatment were equivalent when administered alone or combined with benznidazole treatment to remove the parasite stimulus. Altogether, these data confirm that STAT3 signaling plays a key role in the pathogenesis of CCC.

**Figure 9:**
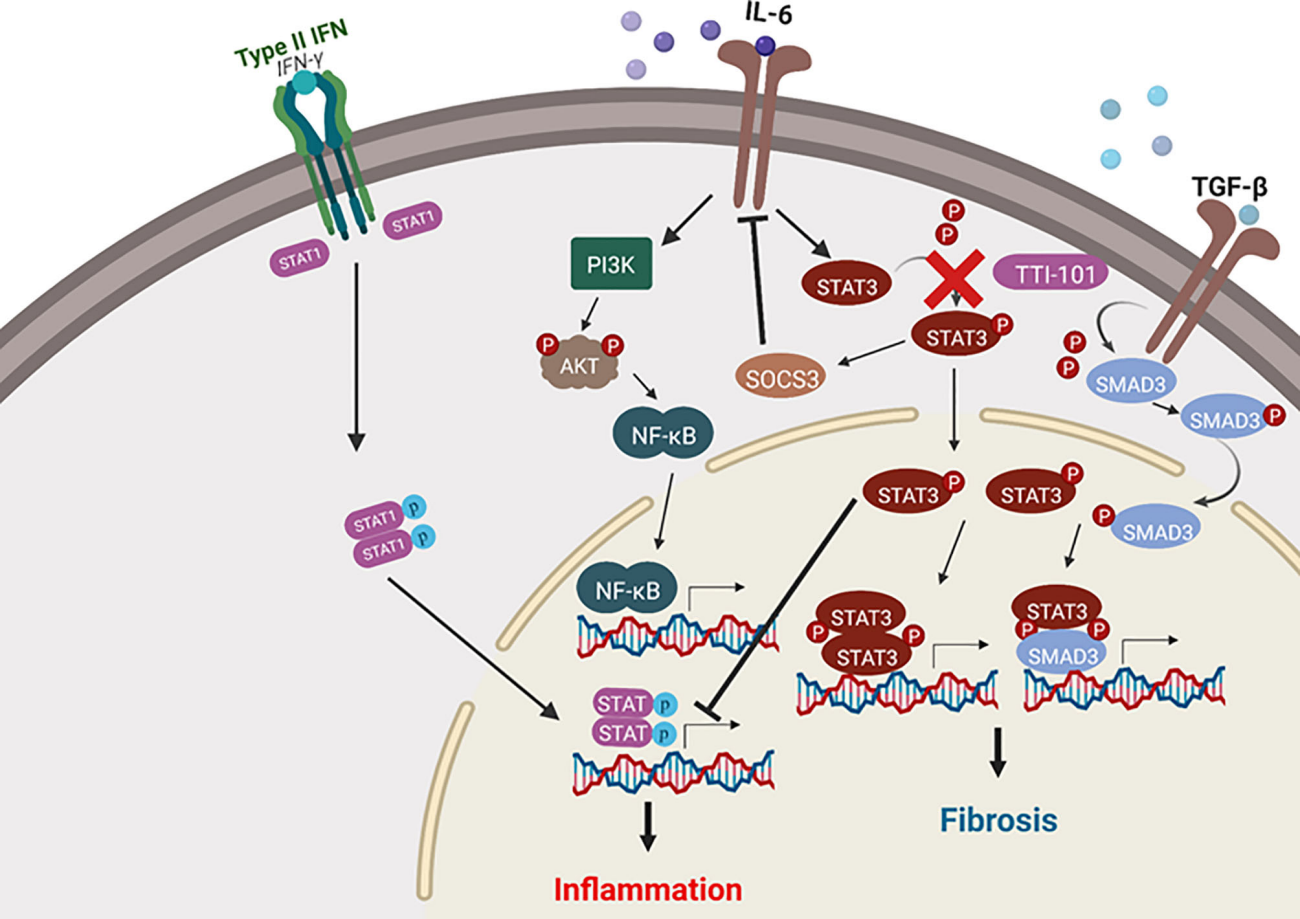
Signal transducer and activator of transcription-3 (STAT3) activity in fibrosis and inflammatory signaling. Depicted are the pro-inflammatory and pro-fibrotic pathways in which STAT3 plays a role. Arrows represent upregulation of a pathway, while blunt lines represent downregulation of a pathway. Cardiac inflammation is modulated by STAT3 *via* suppression of both nuclear factor-κB (NF-κB) and STAT1 pro-inflammatory signaling. Cardiac fibrosis signaling is promoted by STAT3 through transforming growth factor beta (TGF-β)/SMAD signaling. The image was created with the assistance of BioRender.

We propose the pathway illustrated in **Figure 9** as the mechanism of pro-fibrotic communication that is the result of our findings combined with those of previous research, as summarized below (Negoro et al., 2000; Stahl et al., 2013; Stahl et al., 2014; Pedroza et al., 2016; Tang et al., 2017; Cevey et al., 2019). High levels of TGF-β cause increased STAT3 activity through SMAD3 activation and subsequent dimerization between STAT3 and SMAD3, which engage in transcriptional regulation events that promote fibrosis (Tang et al., 2017). The STAT3 activity also has a positive feedback on both TGF-β and PDGF (Negoro et al., 2000; Tang et al., 2017). PDGF stimulates further TGF-β pro-fibrotic signaling (Negoro et al., 2000; Tang et al., 2017; Johnson et al., 2018). Inhibition of phosphorylation of STAT3 would prevent the dimerization and activity of phosphoSTAT3/SMAD3. Ultimately, a positive feedback loop modulated by STAT3 signaling would create an environment of sustained pro-fibrotic signaling between TGF-β and PDGF-D and their downstream gene expression targets, including the collagen gene.

An important finding in our analysis of the cardiac fibrosis development in this study was that benznidazole treatment alone did not reduce cardiac fibrosis, despite successfully reducing cardiac parasite burden. This finding is consistent with results from a 2018 study by Fortes Francisco et al. (2018) demonstrating that benznidazole treatment did not reduce fibrosis in BALB/c mice chronically infected with *T. cruzi*. However, this is in contrast with studies showing that benznidazole treatment does reduce fibrosis in *T. cruzi*-infected C3H/HeJ and C57BL/6 mice (Rial et al., 2020). This suggests that the response to benznidazole treatment may depend in part on the genetic background of the host. Additionally, we found through echocardiographic analysis of heart function that benznidazole treatment did not improve cardiac output or myocardial strain. This is despite the significant reduction in cardiac inflammation in the groups treated with benznidazole, suggesting that benznidazole alone is insufficient to address the underlying host response that leads to clinical disease, similar to what was observed in the BENEFIT trial (Marin-Neto et al., 2009; Morillo et al., 2015).

STAT3 is known to have roles in both pro- and anti-inflammatory signaling, which is tissue and disease dependent (O’Shea and Murray, 2008). As STAT3 inhibition can be immunosuppressive, this may create a situation similar to that in aging immune systems in which the ability to control infection by adaptive immunity is limited and there is subsequent compensation for this deficiency with increased inflammation (Sadighi Akha, 2018). In our model, infection-induced cardiac inflammation was characterized by a dominance of CD8+ T-cell infiltrate into the myocardium. This is consistent with the findings of myocarditis in autopsied human patients with severe CCC, which is also characterized by a dominance of CD8+ T cells in the inflammatory infiltrate (Reis et al., 1993). Accumulation of anti-*T. cruzi* CD8+ T cells in the myocardium in chronic *T. cruzi* infection has been associated with worsened cardiomyocyte lesions, leading to a detrimental outcome for heart function (Lindner et al., 2012; Silverio et al., 2012; Hovsepian et al., 2013; Roca Suarez et al., 2018). Indeed, in our model, the increased inflammatory response after TTI-101 treatment was accompanied by declining cardiac function, further supporting a role for inflammatory damage in decreased cardiac function. This result is similar to previous findings in which STAT3 inhibition worsened cardiac function in other disease models, such as acute myocardial infarction (Negoro et al., 2000). The increase of inflammation, specifically CD8+ T cell infiltrate, shows the importance of the STAT3 pathway in suppressing inflammation in the chronic stage of disease. Our results also align with previous findings that IL-6, in a STAT3-dependent manner, suppresses proliferation and survival of CD8+ T cells in the setting of *T. cruzi* infection (Yu et al., 2013). Future studies should include an investigation into whether these infiltrating CD8+ T cells are *T. cruzi-*specific to evaluate the role of STAT3 in the targeted immune response to the parasite.

Findings from previous research show that both IL-6 and IFN-γ participate in the response to *T. cruzi* infection in animal models (Barry et al., 2016; Jones et al., 2018). Additionally, in human CCC patients, total serum levels of IFN-γ increased later in infection during cardiac dysfunction (Andrade, 1999; Cunha-Neto and Chevillard, 2014). Our results agree with these findings. Furthermore, IL-6 was induced in the TTI-101-treated groups, which we found interesting, as IL-6 is the major ligand for STAT3 signaling and engages in a positive feedback relationship with pY-STAT3. However, recent findings suggest that accumulation of unphosphorylated STAT3 can drive the expression of IL-6 genes through a mechanism distinct from pY-STAT3, suggesting that the IL-6-induced inflammation seen in our results could follow this mechanism (Yang et al., 2007). An important downstream effector of one of these pathways is pro-inflammatory NF-κB, which is induced by IL-6/phosphoinositide 3-kinase (PI3K) signaling (Keller et al., 2016; Jin et al., 2017). Treatment for *T. cruzi* infection with benznidazole has been shown to downregulate NF-κB and Toll-like receptor-4 (TLR4) expression, thereby reducing host inflammatory responses (Ronco et al., 2011; Lambertucci et al., 2017; Cevey et al., 2019). Our findings of increased serum and cardiac IL-6 in the TTI-101-treated group, coupled with increased cardiac gene expression of NF-κB, uncover an IL-6/PI3K/NF-κB pro-inflammatory cascade as a potential important contributor to the intense myocarditis seen in these animals. While there was a slight decrease in inflammatory cell infiltrate in the infected groups treated with benznidazole, there was no decrease in cardiac NF-κB expression. Furthermore, concurrent treatment of benznidazole with TTI-101 abrogated this decrease in inflammation afforded by benznidazole treatment alone. This may be due to other pro-inflammatory mechanisms, such as STAT1-driven inflammation.

In animals treated with TTI-101, IFN-γ was also significantly increased in the serum. IFN-γ is also an important pro-inflammatory cytokine that plays a role in the inflammatory response to *T. cruzi* infection (Rodrigues et al., 1999; Kayama and Takeda, 2010; Rios et al., 2019). STAT3 inhibits IFN-γ/STAT1 and NF-κB pro-inflammatory signaling through various mechanisms, including suppressor of cytokine signaling 3 (SOCS3) upregulation in *T. cruzi* infection, and inhibition of STAT3 results in increased STAT1 activity (Hovsepian et al., 2013; Yu et al., 2017). IL-6-induced STAT3 signaling also likely suppresses IFN pro-inflammatory signaling in CCC. In fibroblasts, IL-6 suppresses IFN-γ/STAT1 induction of pro-inflammatory gene expression in a STAT3-dependent manner (Costa-Pereira et al., 2002). Considering this evidence, combined with the current study’s results, it is clear that STAT3 controls both IL-6/NF-κB and IFN-γ/STAT1 pro-inflammatory signaling, with the overall effect of reducing cardiac inflammation in CCC.

Limitations to this study were present and are acknowledged by the authors. In our previous studies, we evaluated the chronic stage of disease at 111, 140, and 212 DPI, demonstrating persistent low-grade inflammation and progressive fibrosis (Hoffman et al., 2019). In the current study, we evaluated tissue pathology and cardiac function at an earlier time point of 91 DPI. Additionally, we evaluated the impact of benznidazole and TTI-101 treatment immediately at the end of treatment. Since we have shown in our mouse model that, later in disease, cardiac fibrosis levels are higher and cardiac inflammation levels are lower, it is possible that the effects of TTI-101 on cardiac inflammation may be less severe at later stages of CCC. Thus, ongoing studies are evaluating the sustained impact of benznidazole and TTI-101 treatment at later times after infection. Additionally, we observed variability in the reduction of tissue parasite burdens in mice treated with benznidazole, as well as variability in tissue fibrosis levels, with some mice exhibiting higher fibrosis than infected untreated mice. These findings could be the result of treatment failure in individual animals or potentially exacerbated inflammatory responses to parasite antigens leading to increased post-inflammatory fibrosis. Further investigation is needed to identify the causes of the unexpected increases in pathology in individual benznidazole-treated mice.

**Figure 10:**
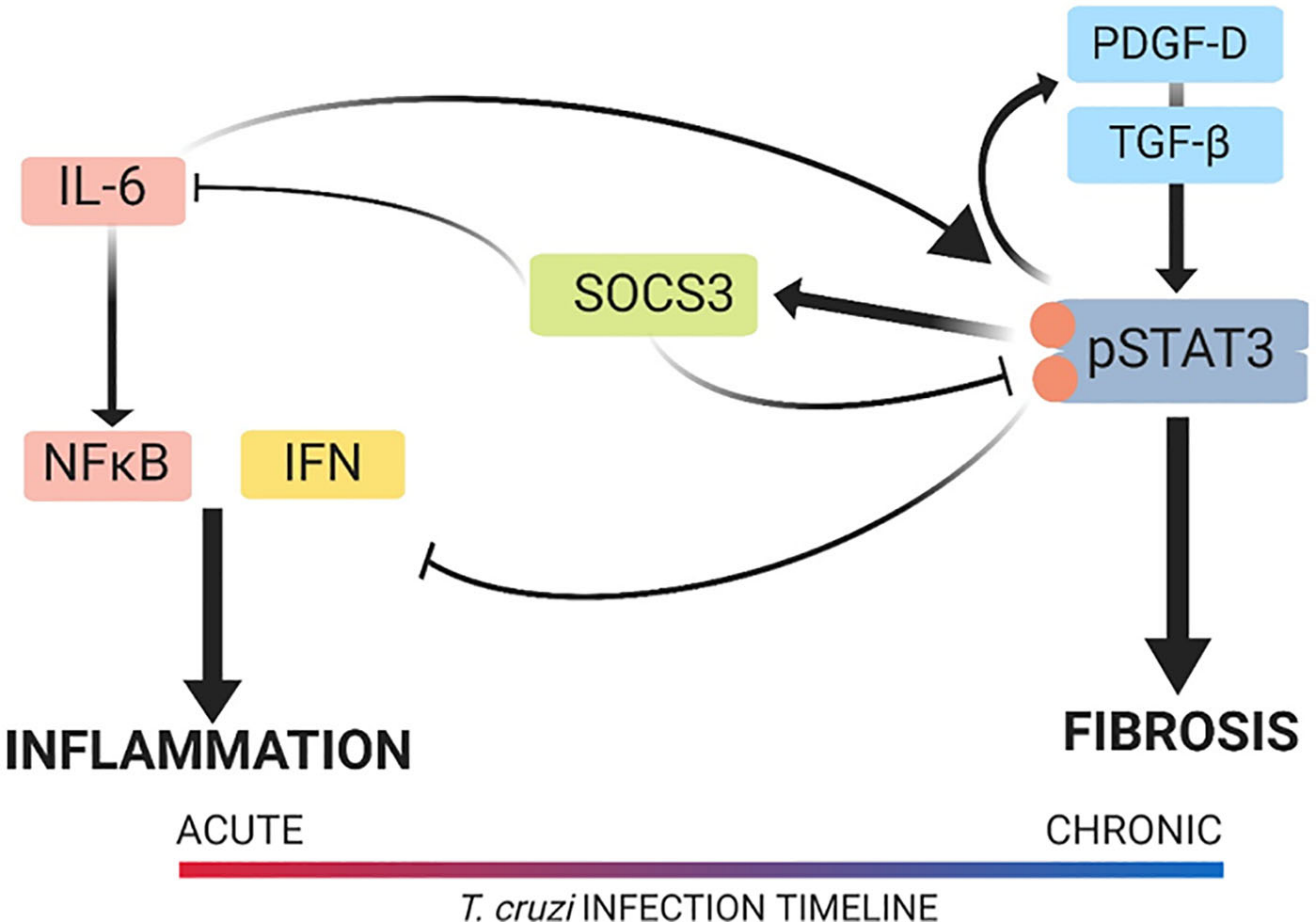
Signal transducer and activator of transcription-3 (STAT3) modulation of the cardiac inflammatory–fibrotic axis in chronic Chagasic cardiomyopathy (CCC). Depicted is the cardiac pathology continuum that starts in the acute phase with a predominantly inflammatory response in the heart, which develops into a predominantly fibrotic response in the heart with progression into chronic disease. The role of STAT3 and its upstream and downstream signaling mediators is also depicted. In our model of CCC, STAT3 was found to promote fibrosis *via* transforming growth factor beta (TGF-β) and platelet-derived growth factor (PDGF) signaling while suppressing inflammatory signaling that involved interferon-γ (IFN-γ) and interleukin-6 (IL-6). Upon inhibition of STAT3, inflammation increased and fibrosis decreased in the heart. The image was created with the assistance of BioRender.

This study uncovered previously unknown signaling pathways responsible for the development of pathology in CCC. Namely, we have described the cellular signaling mechanisms responsible for promoting fibrosis and inhibiting inflammation that involve STAT3, as illustrated in **Figure 10**. The cardiac damage by the parasite and the host inflammatory response create the necessity for a tissue repair mechanism, which, in CCC, is cardiac fibrosis, as seen in our model. STAT3 plays a role in this shift by reducing inflammation and concomitantly promoting fibrosis. This new information illustrates the importance of the balance between pro-inflammatory and pro-fibrotic signaling in the control of cardiac pathology in CCC. The results of this study also suggest that further investigation into the cardiac fibrotic–inflammatory signaling mechanism is warranted for a more complete understanding of the pathophysiology behind CCC. Given recent findings that TGF-β inhibition prevents cardiac fibrosis development, along with our findings that STAT3 inhibition reduces TGF-β-dependent fibrosis development, a more complete understanding of TGF-β signaling in CCC would uncover additional signaling contributors to cardiac pathology. Our findings of the connections between the signaling pathways that induce these pathologies provide insight into the best approach to treatment, which is likely a multimodal therapy to inhibit both inflammation and fibrosis. This new knowledge advances the field by uncovering a new underlying mechanism of disease in CCC.

## Data Availability Statement

The original contributions presented in the study are included in the article/**Supplementary Material**. Further inquiries can be directed to the corresponding author.

## Ethics Statement

The animal study was reviewed and approved by Institutional Animal Care and Use Committee at Baylor College of Medicine.

## Author Contributions

KH, MB, PH, DT, and KJ conceived and designed the experiments. KH, MV, and CP acquired and analyzed the data. KH, DT, and KJ interpreted the data. KH drafted the manuscript. MB, PH, DT and KJ critically revised the manuscript for important intellectual content. KH, MV, CP, MB, PH, DT, and KJ approved publication of the content and agree to be accountable for all aspects of the work. All authors contributed to the article and approved the submitted versión.

## Funding

This work was funded by the Texas Children’s Hospital Center for Vaccine Development to develop and test vaccines for neglected tropical diseases, including Chagas disease. Equipment and reagents used were funded in part by Baylor Research Advocates for Student Scientists. This project was supported by the Mouse Phenotyping Core at Baylor College of Medicine with funding from the NIH (UM1HG006348 and RO1DK114356) and the Pathology and Histology Core at Baylor College of Medicine with funding from the NIH (P30 CA125123). TTI-101 was provided by Tvardi Therapeutics, Inc. 

## Conflict of Interest

DT is the inventor of several patents concerning TTI-101 that are owned by Baylor College of Medicine and licensed to Tvardi Therapeutics, Inc.; DT has ownership of this company’s stock.

The remaining authors declare that the research was conducted in the absence of any commercial or financial relationships that could be construed as a potential conflict of interest.

## Publisher’s Note

All claims expressed in this article are solely those of the authors and do not necessarily represent those of their affiliated organizations, or those of the publisher, the editors and the reviewers. Any product that may be evaluated in this article, or claim that may be made by its manufacturer, is not guaranteed or endorsed by the publisher.

## Footnotes

1 https://www.who.int/en/news-room/fact-sheets/detail/chagas-disease-(american-trypanosomiasis.
