# Supplementary material for: Signal Transducer and Activator of Transcription-3 Modulation of Cardiac Pathology in Chronic Chagasic Cardiomyopathy
Source: Front Cell Infect Microbiol. 2021 Aug 24;11:708325. doi: 10.3389/fcimb.2021.708325 (PMC8421853; doi:10.3389/fcimb.2021.708325)
Supplement: Supplementary file 1 [file DataSheet_1.docx]

**Supplementary Information**

**Title: Signal transducer and activator of transcription-3 modulation of cardiac pathology in chronic Chagasic cardiomyopathy**

**Short Title: STAT3 in chronic Chagasic cardiomyopathy**

**Authors and Affiliations:**

**Kristyn A. Hoffman^1,2^; Maria Jose Villar^2,3^; Cristina Poveda^2,3^; Maria Elena Bottazzi^1,2,3,4^; Peter J. Hotez^1,2,3,4^; David J. Tweardy^5^; Kathryn Jones^1,2,3*^**

^1^ Department of Molecular Virology and Microbiology, Baylor College of Medicine, Houston, TX

^2^ Department of Pediatrics, Section of Tropical Medicine, Baylor College of Medicine, Houston, TX

^3^ Texas Children’s Hospital Center for Vaccine Development, Houston, TX

^4^ Department of Biology, Baylor University, Waco, TX

^5^ Department of Infectious Diseases, Infection Control & Employee Health, Division of Internal Medicine and Department of Molecular & Cellular Oncology, University of Texas MD Anderson Cancer Center, Houston, TX

*Corresponding Author Email [kathrynj@bcm.edu](mailto:kathrynj@bcm.edu)


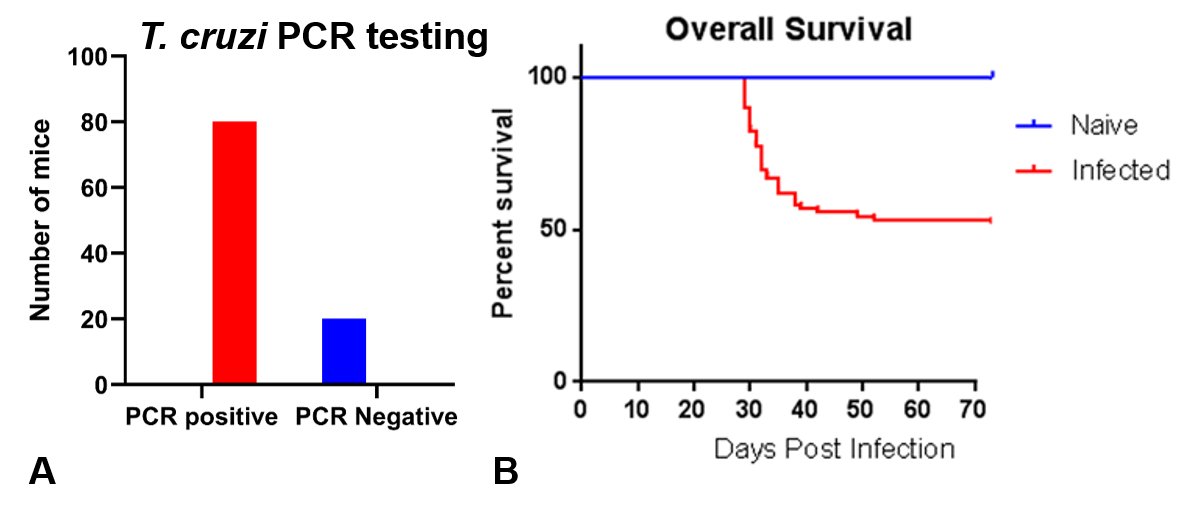


**Supplemental Fig 1**. Acute parasitemia and survival. *T. cruzi* was detected by PCR from blood samples collected 32 DPI from all mice (A). Surival was monitored until 70 DPI (B).
